# Antenatal interventions to support newborn survival: updates for the Lives Saved Tool

**DOI:** 10.7189/jogh.16.04303

**Published:** 2026-10-02

**Authors:** Rahima Yasin, Yvonne Tam, Arjumand Rizvi, Tyler Vaivada, Ayesha Arshad Ali, Robert E Black, Jai K Das, Zulfiqar A Bhutta

**Affiliations:** 1 Institute for Global Health and Development, The Aga Khan University, Karachi, Pakistan; 2 Department of International Health, Bloomberg School of Public Health, Johns Hopkins University, Baltimore, Maryland, USA; 3 Center for Global Child Health, Hospital for Sick Children, Toronto, Ontario, Canada; 4 Department of Paediatrics and Child Health, The Aga Khan University, Karachi, Pakistan

**Keywords:** Lives Saved Tool, antenatal care, neonatal health, vaccination, nutrition, infectious disease

## Abstract

**Background:**

Antenatal care strategies encompass a series of interventions delivered to expectant mothers to optimise both their health outcomes and those of their neonate. We summarise the most recent effect estimates for maternal immunisation, screening and management of infections, pre-existing chronic diseases such as hypertension and diabetes, nutrition, psychosocial interventions for smoking cessation, pre-term pre-labour rupture of membranes, administration of corticosteroids for foetal lung maturation, and induction of post-term labour.

**Methods:**

We synthesised effect estimates for antenatal and intrapartum interventions using PubMed and CENTRAL searches (2022–2023) and in consultation with a technical advisory group to determine whether and how new meta-analysed effect estimates could be incorporated into the Lives Saved Tool (LiST) model, as well as which of these estimates should be updated and which should be retained.

**Results:**

Tetanus toxoid vaccination during the antenatal period lead to a significant reduction in neonatal mortality. Antibiotics for pre-term pre-labour rupture of membranes and administration of corticosteroids for foetal lung maturation lead to significant reductions in neonatal mortality due to sepsis and prematurity, respectively. Nutritional interventions including balanced protein and energy supplementation and multiple micronutrient supplementation reduced the risks of small-for-gestational-age babies. Insecticide-treated bed-nets for malaria, antibiotic treatment of syphilis, and post-term induction of labour demonstrated significant reductions in the risks of stillbirth. The effect estimates for preterm births contributed to the LiST model for interventions including treatment of asymptomatic bacteriuria, omega-3 fatty acid supplementation, calcium supplementation, and provision of low-dose aspirin for pre-eclampsia, which projected significant reductions in the risks of small-for-gestational-age babies.

**Conclusions:**

The evidence described here highlights the potential impact of implementing high-yield healthcare packages in context-specific settings to effectively improve neonatal survival. These estimates can be used as inputs to model the impact of antenatal care interventions using the LiST.

According to United Nations Children’s Fund (UNICEF) estimates, an estimated 1.9 million babies were stillborn in 2023, while 2.3 million infants died within the first month of life [1,2]. Perinatal health outcomes vary significantly between countries and regions, highlighting the need for better resource allocation and access to specialised services [3]. Although statistics, such as maternal mortality relative to neonatal and stillbirth deaths, have improved over the decades, disparities in perinatal healthcare persist, particularly in low- and middle-income countries [4]. The United Nations Sustainable Development Goal 3 calls for reducing neonatal mortality to less than 12 per 1000 live births and the maternal mortality ratio to less than 70 per 100,000 live births, as well as ensuring universal access to all aspects of reproductive healthcare services by 2030 [5].

World Health Organization (WHO) guidelines recommend at least eight contacts to a healthcare provider during the antenatal period to ensure access to essential healthcare interventions for the well-being of mothers and infants [6]. They also endorse a broad range of interventions for improving the utilisation and quality of antenatal care, including nutritional interventions, maternal and foetal assessment, preventive measures, interventions for common physiological symptoms, and health system interventions. Research has also called published evidence-based recommendations to address the multi-faceted challenges of neonatal health by improving quality of care, strengthening health systems, and increasing investments and policy support [7,8].

The Lives Saved Tool (LiST) is a modelling software developed [9] to estimate the potential impact of scaling up coverage of specific maternal, neonatal and child health interventions which could help reduce their mortality and morbidity. To achieve this, it utilises intervention efficacy estimates from meta-analyses to model the impact of the interventions in a consistent and transparent way. It is used by researchers, programme planners, policymakers, and other stakeholders to determine the most effective combination of evidence-based interventions to reduce maternal, neonatal and child mortality rates within a specific setting [10].

In this study, we document the literature derived from existing systematic reviews of interventions and their component trials and informs on the rationale for selecting effect estimates for specific interventions for a range of neonatal outcomes. A related paper examines the evidence for postnatal care interventions to be included in the updated LiST model [11].

## METHODS

We build upon a previously published journal supplement [12–24] by providing a thorough synthesis of evidence on maternal interventions aimed at optimising outcomes for newborn health. We synthesised this evidence using different methodological techniques, which we summarise here and report in detail elsewhere [9,10,24].

### Search and selection of reviews

We conducted a search in PubMed and Central between 2022 and 2023 to identify recent relevant systematic reviews of interventions offered to expectant mothers during the antenatal and intrapartum period, selected for inclusion in the LiST based on prior literature [8] and after consulting the technical advisory group (TAG) and the Johns Hopkins University team that manages LiST (Table S1 in the **Online Supplementary Document**). Details on this procedure are provided elsewhere [12,13,20,24].

The original set of interventions already included in the LiST model represented the foundation for this work [8]; here, we sought to revise these existing estimates (where applicable) using more up-to-date literature. We also identified new interventions that are already being delivered as part of maternal care, but whose effectiveness had not previously been quantified for inclusion in the LiST. The primary requirement was for the effect of antenatal or intrapartum intervention to have a pooled estimate reported for the outcome of neonatal mortality, preterm birth, low birth weight, stillbirth and small-for-gestational-age (SGA) ideally specifically from LMIC studies.

We grouped interventions into two packages, a basic package and an innovation package, to demonstrate the varying levels of feasibility, implementation capacity, and coverage within the LiST model. The basic package included well-established interventions that are backed by strong evidence of effectiveness and are already incorporated into routine maternal and newborn health services. These interventions, potentially scalable, are typically recommended in global guidelines and by the TAG. The innovation package includes interventions which have emerging data and are not proposed in any standard global guidelines. The evidence for these interventions is recent and mostly comes from high-income countries, meaning that they have not been fully tested or implemented in LMICs, but hold promise due to emerging evidence.

To align clinical evidence and pooled estimates with the LiST model, where applicable, we used effects on low birthweight (LBW) to model effects on term SGA, *i.e.* gestational growth, or preterm birth, depending on what was the likely cause of LBW. For interventions such as hypertensive disorder management and diabetes screening, where recent evidence was statistically insignificant or limited, we retained estimates derived from previous Delphi-consensus processes. These retentions were made based on the TAG’s recommendations as a final recourse in our evidence-selection hierarchy to maintain modelling stability for clinically established interventions, where robust systematic reviews or trial data was unavailable, either due to ethical considerations or a lack of high-quality experimental studies. We have flagged these consensus-based parameters in our results to distinguish them from newly updated empirical estimates.

### Quantitative data synthesis

We extracted all relevant data and outcomes with effect estimates after identifying the most recent systematic review. We carried out subgroup analysis for all outcomes of interest reported in studies from LMICs, which we categorised in accordance with the World Bank’s list of countries for the corresponding years when the studies were carried out [25]. When we could not identify interventions from LMICs, we leveraged evidence from high-income or upper-middle income contexts to ensure a comprehensive analysis. We reported pooled statistics as the risk ratio (RR) between experimental and control groups with 95% confidence intervals (CIs). We performed all analyse using RevMan, version 5.1 (Cochrane, London, UK). Based on these effect estimates for relevant outcomes along with other considerations such as regional context and the generalisability of each intervention, we drafted recommendations for inclusion in the LiST model based on the guidelines proposed by Walker *et al*. [9,10] (Table S2 in the **Online Supplementary Document**). We extracted certainty of evidence assessments using the GRADE approach where explicitly reported in the included systematic reviews [26].

## RESULTS

Of the interventions included and modelled, psychosocial interventions to reduce smoking has been added as a new intervention to the LiST model. Omega-3 fatty acids have been modelled as a part of the innovation care package. We retained previous LiST estimates for select interventions, including antibiotics for preterm pre-labour rupture of membranes, management of hypertensive disorders, and screening and management of diabetes. For the latter two interventions, estimates derived from a 2011 Delphi consensus process were maintained to ensure modelling stability. We applied standardised definitions for each intervention to ensure consistency during the modelling process (Table S3 in the **Online Supplementary Document**).

### Interventions initiated during the early antenatal period

#### Tetanus toxoid vaccine

For tetanus toxoid vaccination, we selected a randomised controlled trial (RCT) conducted in Colombia assessing the effectiveness of two or three doses of tetanus toxoid *vs*. influenza vaccine for inclusion in LiST. Tetanus toxoid was associated with a 98% reduction in tetanus-related neonatal mortality (1 study, 688 participants, moderate quality evidence; RR = 0.02; 95% CI = 0.00–0.30) [27]. We propose using this effect estimate for the LiST, which similarly models a near-complete reduction in tetanus-related neonatal mortality as a result of tetanus toxoid vaccination (**Table 1**).

**Table 1:** Early antenatal period

		Quality assessment							Summary of findings				
		**Directness**				**Number of events**							
**Study (year)**	**Review approach**	**Number of studies**	**Outcome**	**Design**	**Limitations**	**Consistency**	**Generalisability to population of interest**	**Generalisability to intervention of interest**	**Intervention**	**Control**	**RR (95% CI)**	**Previous LiST estimate**	**LiST estimate**
**Tetanus toxoid vaccination**													
Demicheli *et al*. (2015) [27]	As-is	1	Neonatal mortality due to tetanus	RCT	Significant effect; fixed effects model used.	Study shows significant benefits; GRADE certainty of evidence: moderate	Study conducted in Colombia (LMIC)	Two or three doses of tetanus toxoid *vs*. influenza vaccine	0 in 341	27 in 347	0.02 (0.00–0.30)	0.94*	0.98
**Treatment of asymptomatic bacteriuria**													
Smaill *et al*. (2019) [28]	As-is	3	Preterm birth	RCT/quasi-experimental RCT	Significant effect	Two studies show benefit; GRADE certainty of evidence: low	All studies are from developed countries	Antibiotics *vs*. no treatment for asymptomatic bacteriuria	9 in 160	29 in 167	0.34 (0.13–0.88)	0.43	0.66 (preterm AGA)
**Prevention of malaria in pregnancy**													
Gamble *et al*. (2007) [29]	As-is	3	LBW	RCT	Insignificant effect	One study shows significant benefit	All studies conducted in LMICs	Provision of ITNs in pregnancy	NA	NA	0.80 (0.64–1.00)	-	0.20 (term SGA)
**Syphilis detection and treatment**													
Tong *et al*. (2023) [30]	As-is	5	Stillbirth	Observational studies	Significant effect; fixed model used	All studies showed consistent benefit	All studies are from LMICs	Two or more doses of penicillin *vs*. at least one dose of penicillin	15 in 545	137 in 531	0.10 (0.06–0.17)	0.82*	0.90
	As-is	7	Preterm birth	Observational studies	Significant effect; fixed model used	Four of seven studies showed consistent benefit	All studies are from LMICs	Two or more doses of penicillin *vs*. at least one dose of penicillin	67 in 612	205 in 539	0.31 (0.24–0.40)	0.51*	0.69 (preterm AGA**)**
**Multiple micronutrient supplementation**													
Keats *et al*. (2021) [31]	As-is	21	Stillbirth	RCT	Significant effect; fixed effects model used	One of twenty-five studies show benefit	All studies are from LMICs	Multiple micronutrient supplementation on *vs*. iron and folic acid or placebo	NA	NA	0.91 (0.85–0.98)	0.09*	0.09
Wang *et al*. (2025) [32]	As-is	14	Term SGA	RCT	Significant effect; fixed effects model used	Three of fourteen studies show benefit	One of fourteen studies is from a developed country	Multiple micronutrient supplementation on *vs*. iron and folic acid or placebo	7321 in 19,326	9155 in 23,292	0.95 (0.93–0.97)	0.05*	0.05
	As-is	14	Preterm SGA	RCT	Significant effect; fixed effects model used	One of twelve studies show benefit	One of twelve studies is from a developed country	Multiple micronutrient supplementation on *vs*. iron and folic acid or placebo	423 in 19326	623 in 23292	0.71 (0.63–0.81)	0.29*	0.29
	As-is	14	Preterm AGA	RCT	Significant effect; fixed effects model used	Two of fourteen studies show benefit	One of fourteen studies is from a developed country	Multiple micronutrient supplementation on *vs*. iron and folic acid or placebo	2638 in 19,326	3144 in 23,292	0.93 (0.89–0.97)	0.07*	0.07
**Balanced protein and energy supplementation**													
Ota *et al*. (2015) [33]	As-is	4	SGA	RCT	Significant effect; fixed effects model used	One of four studies show benefit	All studies are from LMICs	Balanced protein and energy supplementation *vs*. control or no intervention	248 in 1325	290 in 1219	0.80 (0. –0.92)	0.29*	0.2 (term SGA)
		4	Stillbirth	RCT	Significant effect; fixed effects model used	One of four studies show benefit	All studies are from LMICs	Balanced protein and energy supplementation *vs*. control or no intervention	22 in 1489	39 in 1373	0.52 (0.31–0.88)	0.61*	0.48
**Omega-3 fatty acids supplementation**													
Middleton *et al*. (2018) [34]	As-is	26	Preterm birth	RCT	Significant effect; fixed effects model used	One of twenty-six studies shows significant benefit; GRADE certainty of evidence: high	24 of 26 studies are from developed countries	Omega-3 *vs*. no omega-3	608 in 5179	685 in 5125	0.89 (0.81–0.97)	0.10*	0.11 (preterm AGA)
**Calcium supplementation**													
Hofmeyr *et al*. (2018) [35]	As-is	6	Preterm birth	RCT	Significant effect; fixed effects model used	One study showed benefit.	All studies are from LMICs	Routine high-dose calcium supplementation *vs*. placebo in pregnancy	452 in 4957	515 in 5097	0.88 (0.78–0.99)	0.19*	0.12 (preterm AGA)
**Low-dose aspirin provision for pre-eclampsia**													
Hoffman *et al*. (2020) [36]	As-is	1	Preterm birth	RCT	Significant effect	N/A	Study is from LMIC	Aspirin or placebo Tablets	668 in 5780	754 in 5764	0.89 (0.81–0.98)	0.11	0.11 (preterm AGA)
**Psychosocial interventions to reduce smoking**													
Chamberlain *et al*. (2017) [37]	As-is	18	LBW	RCT/quasi	Significant effect; fixed effects model used	No studies show benefit; GRADE certainty of evidence: high	All studies are from developed countries	Psychosocial interventions to reduce smoking in pregnancy *vs*. control	355 in 4743	429 in 4659	0.83 (0.72–0.94)	N/A; modelled as a new intervention (not currently a part of the LiST visualiser)	0.17 (term SGA)

AGA – appropriate for gestational age, CI – confidence interval, LiST – Lives Saved Tool, LMICs – low- and middle-income country, RR – risk ratio, SGA – small for gestational age

*Previous estimates taken from the LiST visualiser.

#### Treatment of asymptomatic bacteriuria

We included this intervention for modelling *via* the list based on consultation with the TAG. Screening and treatment of genitourinary tract infection [28] significantly reduced preterm births in babies born appropriate for their gestational age (3 studies, 320 participants; RR = 0.34; 95% CI = 0.13–0.88) by 66%. All the included studies came from high- or upper-middle income countries. This pooled effect estimate showed a significant magnitude of effect and potential for clinical impact, and we used it to model the effect on preterm (**Table 1**).

#### Prevention of malaria

Insecticide treated bed-nets [29] did not demonstrate a significant impact on LBW (3 studies; RR = 0.80; 95% CI = 0.64–1.00). All trials included were from LMICs and the effect was marginally touching significance; for this reason, we used the LBW estimate in the LiST to model the effect on SGA (20% reduction) (**Table 1**).

#### Syphilis detection and treatment

Tong *et al*. [30] evaluated the effect of two or more doses of penicillin in comparison to at least one dose of penicillin for syphilis in pregnant women. While the systematic review included observational studies from high-, upper-, and lower-middle income countries, we conducted an analysis specifically for LMICs to assess the impact in those regions. Two or more doses of penicillin significantly reduced the risk of stillbirth (5 studies, 1076 participants; RR = 0.10; 95% CI = 0.06–0.17) by 90% and the risk of preterm birth (7 studies, 1151 participants; RR = 0.31; 95% CI = 0.24–0.40) in babies born appropriate for gestational age by 69%. This estimate showed a large magnitude of effect and potential for clinical impact, and we used these effect estimates for LiST to model the impact of detection and treatment of syphilis on stillbirth and preterm birth (**Table 1**).

#### Low-dose aspirin provision for pre-eclampsia

We identified one study [36] which evaluated the effect of low-dose aspirin in pregnant women between gestational ages of 6 weeks and 0 days of pregnancy and 13 weeks and 6 days of pregnancy. It was a large placebo-controlled trial and enrolled participants from six LMICs. Low-dose aspirin supplementation demonstrated a significant effect on preterm birth (1 study, 11,544 participants; RR = 0.89; 95% CI = 0.81–0.98). LiST uses this pooled effect estimate to model the effect of low-dose aspirin supplementation on preterm appropriate-for-gestational age babies (11% reduction) (**Table 1**).

#### Multiple micronutrient supplementation

Keats *et al*. [31] compared the effectiveness of multiple micronutrient supplementation with iron-folic acid supplementation; all the studies included were RCTs, and the former intervention demonstrated a 9% reduction on the risk of stillbirth (21 studies, 96,772 participants; RR = 0.91; 95% CI = 0.85–0.98; 21 studies, n = 96 772). We used these pooled effect estimates in the LiST to model the effect of MMS on term SGA, preterm SGA, and preterm non-SGA (**Table 1**).

We derived effect estimates for outcomes such as SGA and preterm birth from a meta-analysis of individual participant data [32]. Of the fourteen randomised trials included, one was conducted in Mexico (upper-middle income country). Prenatal multiple micronutrient supplementation reduced the risks of term SGA (14 studies, 42,618 participants; RR = 0.95; 95% CI = 0.93–0.97), preterm SGA (14 studies, 42,618 participants; RR = 0.71; 95% CI = 0.63–0.81), and preterm non-SGA (14 studies, 42,618 participants; RR = 0.93; 95% CI = 0.89–0.97) by 5%, 29%, and 7%, respectively. We used this pooled effect estimate in the LiST to model the effect on term SGA and stillbirth (**Table 1**).

#### Balanced protein and energy supplementation

Ota *et al*. [33] evaluated the impact of balanced protein and energy supplementation in pregnant women. We conducted an analysis specifically for LMICs to assess the impact in those regions. All the included studies were RCTs. Balanced protein and energy supplementation demonstrated a reduction in the risks of SGA (4 studies, 2544 participants; RR = 0.80; 95% CI = 0.69–0.92) by 20% and stillbirth (4 studies, 2862 participants; RR = 0.52; 95% CI = 0.31–0.88) by 48%. We used this pooled effect estimate to model the effect on term SGA and stillbirth (**Table 1**).

#### Omega-3 fatty acids supplementation

Omega-3 fatty acids supplementation in pregnancy has been identified by the TAG as a promising new nutrition-based intervention and has been added to the innovation package. Middleton *et al*. [34] assessed the impact of omega-3 fatty acids on preterm birth. RCTs from a mix of high-income, upper-middle income and LMICs were included. The intervention had a significant impact on preterm birth (26 studies, 10,304 participants; RR = 0.89; 95% CI = 0.81–0.97;), leading to an 11% reduction. We used this pooled effect estimate in the LiST to model the effect of omega-3 fatty acids on preterm babies born appropriate for their gestational age (Table 1).

#### Calcium supplementation in at-risk populations

Hofmeyr *et al*. [35] assessed the effect of calcium supplementation in women with low intake of calcium at population level. To assess the effect within LMICs, we conducted a subgroup analysis. The studies utilised a randomised controlled study design. Calcium supplementation during the antenatal period significantly reduced the risk of preterm birth by 12% (6 studies, 10,054 participants; RR = 0.88; 95% CI = 0.78–0.99). We used this pooled effect estimate in the LiST to model the effect of calcium supplementation on preterm babies born appropriate for their gestational age (**Table 1**).

#### Psychosocial interventions to reduce smoking

Chamberlain *et al*. [37] assessed the effectiveness of psychosocial interventions to reducing smoking in pregnant women. The review included RCTs and quasi-RCTs; all included studies were conducted in high-income countries. This intervention is included in the basic package of care and should be available and accessible by all expectant mothers. The intervention demonstrated a significant impact on low birth weight (18 studies, 9402 participants; RR = 0.83; 95% CI = 0.72–0.94). Based on the pooled estimate for LBW, the LiST model estimates a 17% reduction in term SGA babies (**Table 1**).

### Interventions initiated during the late antenatal period

#### Hypertensive disorder case management

In consultation with the TAG, we opted to maintain the previous Every Newborn Action Plan [8] estimates for a 20% reduction in stillbirth applying to both, the antepartum (interquartile range (IQR) = 10–30) and intrapartum (IQR = 10–40) periods, respectively, from the use of antihypertensive agents in pregnancies with mild to moderate hypertension, which was based on the Delphi method (**Table 2**).

**Table 2:** Late antenatal period

		Quality assessment							Summary of findings				
		**Directness**				**Number of events**							
**Study (year)**	**Review approach**	**Number of studies**	**Outcome**	**Design**	**Limitations**	**Consistency**	**Generalisability to population of interest**	**Generalisability to intervention of interest**	**Intervention**	**Control**	**RR (95% CI)**	**Previous LiST estimate**	**New LiST estimate**
**Hypertensive disorder case management**													
								Magnesium sulfate *vs*. placebo				0.2	0.2 (Delphi approach)
								Antihypertensive drugs *vs*. control				0.2	0.2 (Delphi approach)
**Diabetes screening and management**													
								Optimal control *vs*. suboptimal control of diabetes				0.1	0.1 (Delphi approach)
**Antibiotics for pre-term pre-labour rupture of membranes**													
Cousens *et al*. (2010) [38]	As-is	13	Neonatal mortality due to sepsis	RCT	Significant effect; fixed effects model used	Two studies showed benefit; GRADE certainty of evidence: moderate	Ten of thirteen are from developed countries	Antibiotics for pre-term pre-labour rupture of membranes *vs*. control	97	163	0.61 (0.48–0.77)	0.39*	0.39
		13	Neonatal mortality due to prematurity	RCT	Significant effect; fixed effects model used	Two studies showed benefit; GRADE certainty of evidence: moderate	All trials are from developed countries	Antibiotics for pre-term pre-labour rupture of membranes *vs*. control	509	596	0.88 (0.80–0.97)	0.12*	0.12
**Antenatal corticosteroid administration for fetal lung maturation**													
McGoldrick *et al*. (2020) [39]	As-is	5	Neonatal mortality due to prematurity	RCT	Significant effect; random effects model used	Three of five studies show consistent benefit	All studies are from LMICs	Antenatal corticosteroids for preterm labour	312 in 1854	397 in 1837	0.64 (0.43–0.97)	0.15*	0.36

AGA – appropriate for gestational age, CI – confidence interval, LiST – Lives Saved Tool, LMIC – low- and middle-income country, RCT – randomised controlled trial, RR – risk ratio, SGA – small for gestational age

*Previous estimates taken from the LiST visualiser.

#### Diabetes screening and management

In consultation with the TAG, we opted to maintain the previous 2014 Every Newborn Action Plan [8] estimate, where experts used the Delphi approach due to paucity of data from LMICs and determined a 10% reduction in stillbirth with optimal control of diabetes during the antepartum (IQR = 5–30) and intrapartum (IQR = 3.5–25) periods (**Table 2**).

#### Antibiotics for pre-term pre-labour rupture of membranes

Cousens *et al*. [38] evaluated the effectiveness of antibiotics for preterm pre-labour rupture of membranes based on data from RCTs. The intervention led to a 39% reduction in the risk of neonatal mortality due to sepsis (13 studies; RR = 0.61; 95% CI = 0.48–0.77), which we used in LiST. Additionally, the use of antibiotics for pre-term pre-labour rupture of membranes also demonstrated an effect on neonatal mortality due to prematurity (13 studies; RR = 0.88; 95% CI = 0.80–0.97). We used this 12% reduction in deaths from prematurity in the LiST (**Table 2**).

#### Antenatal corticosteroid administration for foetal lung maturation

McGoldrick *et al*. [39] evaluated the effect of antenatal corticosteroids for accelerating lung maturation in women at risk of preterm birth. All of the studies were RCTs. We conducted an analysis specifically for LMIC to assess the impact in those regions. Antenatal corticosteroids had a significant 36% reduction in neonatal mortality (5 studies, 3691 participants; RR = 0.64; 95% CI = 0.43–0.97). We used this effect estimate in the LiST to model the effect of antenatal corticosteroids on neonatal mortality due to prematurity (**Table 2**).

### Interventions for the intrapartum period

#### Post-term induction of labour

Middleton *et al*. [40] evaluated the effect of inducing labour in pregnancies lasting over 41 weeks and identified a 76% reduction in the risk of stillbirth (15 studies, 10,909 participants; RR = 0.24; 95% CI = 0.08–0.78). All included studies were RCTs from a mix of high-, middle-, and lower-middle income countries with a potential for clinical impact. We used this effect in the LiST (**Table 3**).

**Table 3:** Intrapartum period

		Quality assessment							Summary of findings				
		**Directness**				**Number of events**							
**Study (year)**	**Review approach**	**Number of studies**	**Outcome**	**Design**	**Limitations**	**Consistency**	**Generalisability to population of interest**	**Generalisability to intervention of interest**	**Intervention**	**Control**	**RR (95% CI)**	**Previous LiST estimate**	**New LiST estimate**
**Post-term induction of labour**													
Middleton *et al*. (2020) [40]	As-is	15	Stillbirth	RCT	Significant result. Fixed model used.	No studies show benefit	Ten of fifteen studies are from developed countries	Induction of labour for pregnancies lasting ≥41 weeks	1 in 5472	12 in 5437	0.24 (0.08–0.78)	0.7*	0.76

CI – confidence interval, LiST – Lives Saved Tool, RCT – randomised controlled trial, RR – risk ratio

*Previous estimates taken from the LiST visualiser.

## DISCUSSION

In this review, we evaluated 15 interventions implemented during the early and late antenatal period, and the intrapartum period, deriving the pooled effect estimates from clinical data within existing systematic reviews and meta-analyses. Our selection of the most recent systematic reviews, guided by consultations with the TAG, was based not solely on publication date, but also on a hierarchal assessment of methodological quality and the comprehensiveness of the review’s search strategy. Where possible, we derived the effect sizes from several robust randomised controlled trials, but such high-quality data were not consistently available for mortality outcomes for interventions such as treatment of bacteriuria and syphilis, which further emphasises the need for strengthened research efforts into this topic. Based on the TAG’s recommendations, we opted to maintain the previous 2014 Every Newborn Action Plan evidence base for interventions such as hypertensive case disorder management and diabetes screening and management, which employed pre-existing Delphi consensus estimates. For the management of preterm pre-labour rupture of membranes, we used historical studies to generate estimates, given the limited evidence from LMICs and ethical considerations, such as offering antibiotics to expectant mothers *vs*. providing little or no medical care, especially in low-resource settings.

While the LiST model estimates the potential impact of scaling up coverage of specific maternal health interventions which could help reduce maternal, neonatal and child mortality, morbidity and stillbirths, it has some inherent applicability and feasibility constraints for LMICs. For example, contextual differences such as baseline risk profiles, high comorbidity burdens, and variations in treatment quality in low-resource settings may alter real-world effect sizes relative to pooled estimates drawn from high-income settings. Furthermore, LMICs often face bottlenecks in their health systems, including limited referral capacity and reduced access to diagnostic infrastructure. Implementation in these settings, therefore, requires careful consideration of these contextual factors to ensure that scaling up coverage of specific interventions is sufficiently robust to deliver adequate neonatal care.

Psychosocial interventions to reduce smoking were newly added to the LiST model. These interventions leverage counselling, health education, financial incentives, and social support frameworks to reduce the risk of LBW. While the current evidence base relies on trials conducted in high-income countries [37], we believe the LiST model can still effectively project the impact of these interventions in LMICs. Additionally, while the evidence for omega-3 fatty acid supplementation demonstrating a significant reduction in preterm birth is mostly derived from high-income settings, it serves as a potentially effective and promising ‘innovative’ intervention for future modelling in LiST. However, additional trials in LMICs are required to warrant inclusion within the basic LiST package.

Evidence from LMICs was unavailable for interventions such as treatment of asymptomatic bacteriuria, psychosocial interventions to reduce smoking, hypertensive disorder case management, and screening and management of diabetes. To bridge this gap, we leveraged effect estimates from high- and upper-middle-income countries and the Delphi consensus. For interventions including omega-3 fatty acids supplementation, management of preterm pre-labour rupture of membranes and induction of labour, we utilised pooled data from a mix of high- and upper-middle income countries and LMICs.

Several studies [41–43] have assessed the optimal doses and time to administer the tetanus toxoid vaccine during pregnancy at which the maternal immune response and placental antibodies’ transfer is most efficient, providing a clear rationale for reinforcing vaccination requirements within the basic antenatal care packages. Serra *et al*. [44] and Sun *et al*. [45] did not find a reduced risk of preterm birth after omega-3 supplementation in pregnancy, but Abdelrahman *et al*. [46] observed decreases in risks of preeclampsia, low birthweight, and preterm birth. Romero *et al*. [47] and Conde-Agudelo *et al*. [48] reported decreased risks of preterm birth in singleton gestations with mid-trimester sonographic short cervix, but recommended more investigations on other perinatal outcomes. Strong evidence indicates multiple micronutrient supplementation improves key outcomes like preterm birth, SGA, and LBW compared to iron and folic acid supplementation [49], which is consistent with our findings. Chen *et al*. [50] compared low-dose aspirin combined with calcium supplementation to aspirin alone and reported the former to be more effective in prevention of preeclampsia and reduction of risk of preterm birth. Other reviews [51–53] also reported lower rates of preeclampsia with calcium supplementation, which is consistent with our results. However, more investigations are needed to determine the ideal dose for this intervention. Antenatal corticosteroids were reported by Ciapponi *et al*. [54] to be effective for improving neonatal outcomes among women at risk of preterm birth, with no significant differences in efficacy observed between different corticosteroid regimens. Simultaneously, Ninan *et al*. [55] found scarce data for comparing lower doses to standard double doses. Antibiotics for the management of preterm pre-labour rupture of membranes seem to be more effective than placebo in reducing the rate of chorioamnionitis, but Chatzakis *et al*. [56] reported that no single antibiotic is more superior to another, however the evidence is of low quality. Alkmark *et al*. [57] assessed induction of labour at 41 weeks and found improved perinatal outcomes compared with expectant management until 42 weeks in nulliparous women only. The interventions delivered in this research, however, are contextual and their quality varies across different regions, which affects the extent to which coverage translates into lives saved. Overall, our findings indicate that the implementation of the basic care package during the antenatal and the intrapartum period has the potential to translate to real-world impact.

## CONCLUSIONS

We summarised the most recent and up-to-date evidence on a diverse range of essential interventions given to expectant mothers during the antenatal and the intrapartum period and subsequently synthesised the impact of a package of effective antenatal and intrapartum interventions for neonates for an updated LiST model. These updated effect estimates allow for a more precise estimation of how scaling up intervention coverages in LMICs can influence neonatal survival.

## Additional material


Online Supplementary Document
