# Supplementary material for: Antenatal interventions to support newborn survival: updates for the Lives Saved Tool
Source: J Glob Health. 2026 Oct 2;16:04303. doi: 10.7189/jogh.16.04303 (PMC13631934; doi:10.7189/jogh.16.04303)
Supplement: Supplementary file 1 [file jogh-16-04303-s001.pdf]

Supplement to: Yasin R, Tam Y, Rizvi A, Vaivada T, Ali AA, Black RE, Das JK, Bhutta ZA. Antenatal interventions to support newborn survival: updates for the Lives Saved Tool. J Glob Health. 2026;16:04303.

Table S1. Conceptual Framework for Maternal Care Interventions

Early Antenatal Care Interventions	Late Antenatal Care Interventions	Intrapartum Care Interventions
• Tetanus toxoid vaccination<br>• Treatment of asymptomatic bacteriuria<br>• Prevention of malaria in pregnancy<br>• Syphilis detection and treatment<br>• Multiple micronutrient supplementation<br>• Balanced protein and energy supplementation<br>• Omega 3 fatty acids in pregnancy<br>• Calcium supplementation in women with low intake of calcium at population level<br>• Low-dose aspirin for prevention of pre-eclampsia in high-risk pregnancies<br>• Psychosocial interventions to reduce smoking	• Hypertension disorder case management<br>• Diabetes case management<br>• Antibiotics for pre-term pre-labour rupture of membranes<br>• Antenatal corticosteroids for fetal lung maturation	• Post-term induction of labor

Table S2. Summary of possible considerations for recommendation of a maternal intervention for scale-up and inclusion in the Lives Saved Tool based on available evidence

Evidence Category	Considerations, Explanations, and Examples
Outcome and pooled effect estimate	
Magnitude and significance of effect estimate	The strength of the effect and potential for clinical relevance or impact at the population

Nature of outcome	Is the outcome related to mortality (all-cause or cause-specific), stillbirth or small-for-gestational age (SGA)?<br>Can the outcome be considered a risk factor (e.g., preterm birth, low birthweight), or is it a direct measurement of a clinical endpoint (e.g., mortality, SGA)?<br>If there is no measured or insufficient effect on a clinical endpoint, can a recommendation still be made when there is a strong effect on a key risk factor?
Width of 95% confidence interval	Indicator of certainty of the effect estimate
Total sample size of pooled estimate	Indicator of reliability of the effect estimate
Total number of studies included in pooled estimate	Higher number of studies allows better assessment of potential consistency of effect across different settings and populations
Included studies and trials	
Design of included studies	More robust quality study designs (e.g., RCT) contribute to higher strength of evidence
Methodological quality and risk of bias of included studies	Lower assessed risk of bias contributes to higher strength of evidence
Heterogeneity of included studies	Indicator of the consistency of the effect across settings and populations
Results of subgroup and/or sensitivity analyses	Allows for the determination of effect differences between settings, populations, or other measurable characteristics of the intervention
Applicability of evidence to the LMIC context	
LMIC-specific evidence available	Provides evidence for effectiveness that is more aligned with recommendations for resource-constrained settings
Proportion of LMIC-specific evidence	Proportion of evidence that is more aligned with recommendations for resource constrained settings
Potential application and feasibility of intervention in LMIC setting	Pragmatic assessment of the real-world potential for implementation and scale up of the intervention in resource constrained settings
Ability to leverage, and applicability of, HIC evidence where LMIC data unavailable	Pragmatic assessment of the comparative burden of disease and expected variation in the actual effect of an intervention between HIC and LMIC settings

Table S3. Names and definitions of antenatal interventions included in the Lives Saved Tool.

Intervention Name	Definition	Package
**Early Antenatal Care**		
Tetanus toxoid vaccination	Percent of neonates who are protected at birth (PAB) from tetanus infection. PAB is defined as the percent of women who received two doses of tetanus toxoid during this pregnancy or ever: Received at least 2 doses, the last within 3 years; received at least three doses, the last within 5 years; received at least 4 doses, the last within 10 years; or received at least five doses during lifetime. Also known as TT2+.	Basic
Treatment of asymptomatic bacteriuria	Percent of pregnant women receiving therapeutic antibiotics for initial treatment of serious infections of the pelvic organs or upper urinary tract	Basic
Prevention of malaria in pregnancy	Percent of pregnant women receiving 2+ doses of Sp/Fansidar or sleeping under an long-lasting insecticide treated net (LLIN) during pregnancy. Coverage is calculated as the greater of either a) IPTp coverage or b) coverage of LLIN-use among pregnant women based on household survey data.	Basic
Syphilis detection and treatment	Percent of pregnant women tested for syphilis and given treatment if needed.	Basic
Multiple micronutrient supplementation	Percent of pregnant women taking a multiple micronutrient supplement daily. A multiple micronutrient supplement is defined as a supplement containing at least iron, folate, and additional vitamins/minerals.	Basic
Balanced protein and nutrient supplementation	Percent of pregnant women who are food insecure who receive balanced protein energy supplementation.	Basic
Omega 3 fatty acids supplementation	Percent of pregnant women who receive omega-3 fatty acids supplement	Innovation
Calcium supplementation in women with low-intake of calcium at population level	Percent of pregnant women who are calcium deficient	Basic
Low-dose aspirin provision for the prevention of pre-eclampsia in high-risk pregnancies	Percent of pregnant women at risk of preterm births who receive low-dose aspirin	Basic

Psychosocial interventions to reduce smoking	Women who are currently smoking or have recently quit smoking	Basic
**Late Antenatal Care**		
Hypertension disorder case management	Percent of pregnant women receiving detection and appropriate management of moderate to severe hypertension during pregnancy.	Basic
Diabetes case management	Percent of pregnant women screened for diabetes and managed appropriately, if needed.	Basic
Antibiotics for preterm or prolonged premature rupture of membranes	Percent of pregnant women with premature rupture of the membranes (pPRoM) who are not in labor and receive prophylactic antibiotics.	Basic
Antenatal corticosteroids for fetal lung maturation	Percent of women with premature labor receiving an intramuscular injection of betamethasone sodium phosphate (6mg, every 12 hours for 2 days).	Basic
**Intra-partum Care**		
Post-term induction of labor	Percent of women who are 41 or more weeks pregnant who are managed with induction of labor as appropriate	Basic
